# Retraction: Extract of *Spatholobus suberctus* Dunn ameliorates ischemia-induced injury by targeting miR-494

**DOI:** 10.1371/journal.pone.0212836

**Published:** 2019-02-20

**Authors:** The PLOS ONE Editors

Following the publication of this article [1], concerns were raised that background pixelation patterns are highly similar between individual Western blot panels in each of the relevant figure panels.

The authors have not responded to the journal’s queries and we have not received a response from the corresponding author’s institution (Affiliated Hospital of Zunyi Medical College) regarding the concerns raised.

Furthermore, the underlying data supporting the results in this article have not been provided, per our Data Availability policy.

In light of the image concerns and the unavailability of underlying data, the *PLOS ONE* Editors retract this article.

The authors did not comment on the retraction decision.
